# Treatment outcomes, symptoms of anxiety and depression, and satisfaction with life after orthognathic surgery

**DOI:** 10.3389/froh.2026.1835432

**Published:** 2026-05-25

**Authors:** Kine Jespersen, Thomas Brox, Sølve Hellem, Bo Wold Nilsen, Ellen Nordal, Jan-Are K. Johnsen, Sigurd Hadler-Olsen, Paula Frid

**Affiliations:** 1 Public Dental Health Service Competence Centre of North Norway, Tromsø, Norway; 2 Department of Otorhinolaryngology, Division of Oral and Maxillofacial Surgery, University Hospital North Norway, Tromsø, Norway; 3 Department of Clinical Dentistry, University of Bergen, Bergen, Norway; 4 Department of Clinical Dentistry, UiT The Arctic University of Norway, Tromsø, Norway; 5 Department of Clinical Medicine, UiT The Arctic University of Norway, Tromsø, Norway; 6 Department of Pediatrics and Adolescence Medicine, University Hospital of North Norway, Tromsø, Norway

**Keywords:** HADS, orthognathic surgery, PAR-index, QOL, SWLS

## Abstract

**Background:**

Psychological factors may affect surgical outcomes. This study aimed to evaluate the treatment results of orthognathic surgery, and to investigate how anxiety, depression and satisfaction with life affect overall patient satisfaction.

**Methods:**

Data was collected preoperatively and median 28 months postoperatively at a single center in 2016–2025. Treatment outcomes were assessed using The Peer Assessment Rating (PAR) index and cephalometric data, and complications were recorded. The Hospital Anxiety and Depression Scale (HADS), and the Satisfaction with Life Scale (SWLS) were used for psychological assessment. Satisfaction with treatment was assessed on a 5-point Likert scale.

**Results:**

Twenty-four patients were included in the study, all of whom underwent preoperative and postoperative psychological consultations. The majority of the participants were female (83%), with a median age (IQR) of 23 years (20–34). Dental occlusion, i.e., PAR-index score improved with 86% but did not correlate with satisfaction with treatment. Most cephalometric variables were normalized. Complications were common, occurring in 12 out of 24 cases, but were generally mild in severity. HADS and SWLS were within normal range and did not change significantly postoperatively. HADS did not correlate to satisfaction with treatment, however SWLS preoperatively correlated negatively with postoperative satisfaction with dental occlusion (*p* = 0.020). Most patients were satisfied with all aspects of treatment (61%–86%), except for the overall duration and the healing period.

**Conclusions:**

The study results underline the importance of comprehensive preoperative assessment and specific information regarding healing time, total treatment duration, and potential complications. This approach may enhance patient satisfaction and identify individuals who require additional support throughout the treatment process.

## Introduction

Orthognathic surgery is an established treatment option for 1%–5% of the population with severe dentofacial discrepancies (1, 2), aiming to improve chewing function and facial appearance. While dentofacial deformities may be associated with juvenile idiopathic arthritis (JIA) (3), sleep apnea (4) or craniofacial syndromes (5), most patients are healthy. The treatment is, despite its benefits, demanding and carries certain risks. Patient cooperation is crucial to obtain a positive outcome, and it is therefore important to have information about the patients' expectations and thoughts concerning the treatment (6). Also, it is important that patients are informed about risks and variations in outcome and what factors that can affect these variations (7). Studies have indicated that orthognathic surgery may yield positive outcomes in terms of psychosocial well-being and self-concept (8, 9), and that orthognathic patients overall are satisfied with the treatment results (10). Patient satisfaction is however influenced by various factors, including motivation, expectations, psychological status, and social support (11). Depressive symptoms are known to be associated with non-compliance to medical treatment and to treatment outcome in general (12), and also associated with higher levels of postoperative pain (13). This emphasizes the importance of assessing these symptoms to identify patients that might need more support to optimize treatment outcome and satisfaction. Therefore, in some countries, for instance the UK, it is common to do a psychological evaluation before orthognathic surgery (14). This is however not an established routine in Norway. To assess psychological factors, validated psychometric tools (15, 16) can be utilized. However, few studies on orthognathic surgery evaluating treatment outcomes, have used validated psychometric tools nor evaluated how depression and anxiety affect the degree of satisfaction.

The aim of this study was therefore to evaluate treatment outcomes following orthognathic surgery, with a focus on dental occlusion, skeletal deformities, complication rates, and to examine how psychological factors influence patient satisfaction in individuals undergoing orthognathic surgery.

## Materials and methods

### Study design and patients

This prospective observational study used data from patients assessed for orthognathic surgery at the Public Dental Health Service Competence Centre of North Norway (TKNN) and who underwent orthognathic surgery at the University Hospital of North Norway (UNN) in the period of 2016–2025. *Inclusion criteria* were patients with severe dentofacial deformities assessed and discussed in the local Expert Group for Orthognathic Surgery, and with informed consent who had orthognathic surgery with or without orthodontic treatment, and psychological consultation before and after surgery. *Exclusion criteria* were patients with craniofacial syndromes, which according to guidelines are treated at a national center.

### Clinical assessments

Patients were consecutively assessed by a specialist in oral and maxillofacial surgery, an orthodontist and a psychologist before and postoperatively minimum 12 months after orthognathic surgery. Data was collected from medical records and included demographics, surgical procedures (Le Fort 1 osteotomy, bilateral sagittal split osteotomy (BSSO), vertical ramus osteotomy (VRO), genioplasty, and bimaxillary surgery), follow-up time after surgery, medical history, medication use, and use of tobacco. Complications after surgery were classified according to the Serious Adverse Event (SAE) criteria (17). Preoperative and/or postoperative study casts (plaster and/or digital models) were analyzed by an orthodontist using the Per Assessment Rating Index (PAR-index) (18). The PAR-index is an objective orthodontic instrument used to measure the severity of dental malocclusion and evaluate the success of orthodontic treatment. It calculates a single summary score by assessing pre- and post-treatment scores. The outcome of treatment was assessed using the PAR index nomogram as well as calculation of percent improvement: 1, Great improvement >70%; 2, Improvement 30%–70%; 3, Worse or no difference 0%–30%.

Cephalometric analyses were performed using the Facad® software program for orthodontic tracing. The following landmarks were recorded: ANB, the angle formed by point A, nasion (N) and point B; Wits appraisal, the distance between point A and point B projected perpendicularly onto the occlusal plane; ML-NSL, the angle formed by the Mandibular line and Nasion-Sella-Line; Li-NB, the inclination of the lower incisors in relation to the nasion-point B line and Li-ML, Lower Incisor to the Mandibular Plane.

### Psychological assessments

Patients were assessed by a psychologist before and minimum 12 months after orthognathic surgery and they were asked to complete the Norwegian version of the following questionnaires: the Hospital Anxiety and Depression Scale (HADS) (15), and the Satisfaction With Life Scale (SWLS) (16). The HADS consists of 14 items with four response alternatives, measuring anxiety and depressive symptoms on a scale from 0 to 42 (higher score indicating more anxiety and depression). The HADS also gives separate scores for anxiety and depression, but in this study the HADS-total was used to provide a single, straightforward measure of emotional distress. The goal was to identify individuals experiencing psychological distress, regardless of whether it was primarily anxiety or depression, and how this affects the treatment outcome of orthognathic surgery. The total score serves as a broad screening tool for this purpose.

There is no universally agreed-upon cutoff for the HADS-total score. The SWLS is a five-item scale where individuals rate the extent to which they agree or disagree with certain statements on a scale from 1 to 7 (1 = strongly disagree, 7 = strongly agree) with a total score ranging from 5 to 35. Higher scores indicate greater life satisfaction: 31–35 “Extremely satisfied”, 26–30 “Satisfied”, 21–25 “Slightly satisfied”, 20 “Neutral”, 15–19 “Slightly dissatisfied”, 10–14 “Dissatisfied”, 5–9 “Extremely dissatisfied” (16).

Patients also completed a study specific questionnaire (i.e., not based on any international validated scales) to investigate satisfaction with the outcome and process (facial aesthetics, dental occlusion, TMJ-function, healing time, total treatment time) and with the psychological assessment on a 5-point Likert scale: very satisfied, satisfied, neutral, less satisfied, dissatisfied.

## Ethics

The Regional Committee for Health Research Ethics (REK) was consulted and concluded that the study was a quality assurance study (Reference number 2751) and therefore did not need an approval. The study was conducted in accordance with the Declaration of Helsinki with informed, written consent from all participants. The local data protection authority at UNN approved the study.

## Statistics

Descriptive statistics were used to summarize demographics, clinical and radiological variables, including medians (interquartile ranges), means (standard deviations), and frequencies (percentages). Paired t-tests were applied to compare HADS and SWLS scores, PAR-index, and cephalometric data before and after orthognathic surgery for normally distributed data, while the Wilcoxon signed-rank test was used for non-normally distributed data. Correlations (Spearman's rho) were used to analyze associations between scores in HADS/SWLS before surgery and patient satisfaction with treatment outcomes and process.

## Results

### Patients, demographics and clinical characteristics

**Figure 1:**
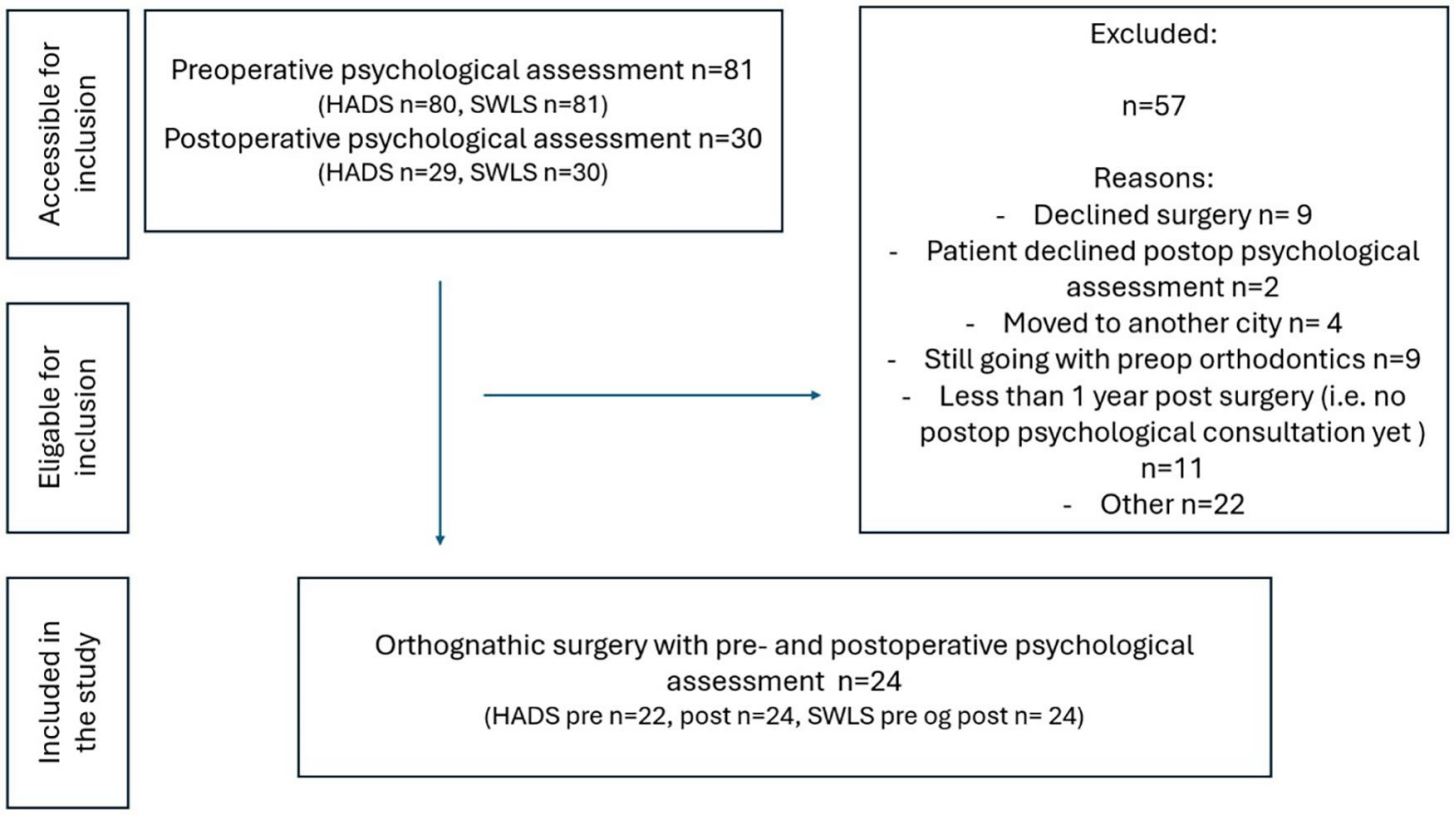
Flowchart of patients going through orthognathic surgery assessments and psychological assessments at the Public Dental Health Service Competence Centre of North Norway in 2016–2025. Adapted from “PRISMA 2020 flow diagram template for systematic reviews” by Page et al., licensed under CC BY 4.0.

Twenty-four patients were included in the study with both preoperative and postoperative clinical and psychological assessments among the 81 patients that had an initial preoperative psychological assessment at TKNN in the period 2016 to 2025 (Figure 1, flowchart). The demographics and clinical characteristics are reported in Table 1. There was a female predominance (83%), and the median age at orthognathic surgery was 23 years.

**Table 1:** Clinical characteristics of patients who underwent orthognathic surgery at the University Hospital of North-Norway 2016–2025 (*n* = 24).

	*n* (%)
Demographics	
Female	20 (83)
Ethnicity	
Norwegian/Caucasian	23 (96)
Age at orthognathic surgery assessment, years	21 (18–33)
Age at surgery, years	23 (20–34)
Time between preop psychological consultation and surgery, months	18 (10–24)
Time between surgery and postop psychological consultation, months	28 (21–38)
Comorbidities	
Comorbidities	
Lung disease (including asthma)	3 (13)
Cardiovascular disease	1 (4)
ADHD	2 (8)
Migraine	2 (8)
Mental disorder	4 (17)
Mild mental retardation	1 (4)
JIA/RA	3 (13)
Use of tobacco	5 (21)

Values are the median (1st to 3rd quartile) unless indicated otherwise. ADHD, attention deficit hyperactivity disorder; JIA, juvenile idiopathic arthritis; RA, rheumatoid arthritis.

### Diagnoses, surgical procedures and complications

Diagnoses, surgical procedures and complications are reported in Table 2. The distribution of basal sagittal relations (Angle class I, II and III) were 43/24/33 percent. Vertically, 38/24/38 percent had neutral/deep/open basal relations and, transversally approximately 17% of the patients had a facial asymmetry. Single jaw orthognathic surgery (58%) was more common than bimaxillary jaw surgery. Complications were frequent (*n* = 12/24 patients) and generally mild. However, one patient experienced a severe infection despite having discontinued immunosuppressive medication prior to surgery. This was classified as a Serious Adverse Event (SAE), which resulted in prolonged hospitalization. The patient required surgical debridement for bone sequestration, hyperbaric oxygen therapy, intravenous antibiotic treatment, and removal of the osteosynthesis plate. One patient reported a neurosensory disturbance, i.e., altered sensation in her lower lip. Another patient received a bad split during a BSSO osteotomy with limited effect on the dental occlusion. Simple infections (*n* = 1) were treated with antibiotics, while deviated nasal septum was either accepted by the patient (*n* = 1) or was corrected through a nasal septoplasty procedure (*n* = 2). Necrotic teeth (*n* = 3) were treated with endodontic treatment (i.e., root canal filling). Two patients had early skeletal relapses and were treated with prolonged postoperative orthodontic treatment. TMD (*n* = 1) were treated with botulinum toxin and gingival retractions (*n* = 1) with gingival plastic procedure. One patient with persisting asymmetry was offered another genioplasty but preferred to avoid further surgery. Exhaustion and weight problems were reported in one patient after orthognathic surgery and was handled by the local physician.

**Table 2:** Diagnoses, surgical procedures and complications in patients (*n* = 24) going through orthognathic surgery at the University Hospital of North-Norway in 2016–2025.

	*n* (%)	*n*
Diagnosis sagittal relation		
Class I	9 (43)	21
Class II	5 (24)	21
Class III	7 (33)	21
Diagnosis vertical relation		
Neutral	6 (38)	16
Deep	6 (38)	16
Open	4 (25)	16
Diagnosis facial asymmetry	4 (17)	24
Orthognathic surgery procedures		
Le Fort 1	5 (21)	24
BSSO	6 (25)	24
Bimax	6 (25)	24
Bimax + Genioplasty	4 (17)	24
BSSO + Genioplasty	1 (4)	24
Le Fort 1 3-piece maxilla	2 (8)	24
Segmented maxilla	6 (25)	24
Complications		
Neurosensory disturbance lower lip	1 (4)	24
Bad split	1 (4)	24
Serious infection/bone sequestrum	1 (4)	24
Simple infection	1 (4)	24
Deviated nasal septum	3 (13)	24
Necrotic tooth	3 (13)	24
Early skeletal relapse	2 (8)	24
TMD	1 (4)	24
Gingival retractions	1 (4)	24
Persisting asymmetry	1 (4)	24
Exhaustion and weight problems	1 (4)	24

BSSO, bilateral sagittal split osteotomy; Bimax, bimaxillary osteotomy.

### PAR-index and satisfaction with treatment

There was a significant improvement (*P* < 0.001) in dental occlusion measured by the PAR-index with a change of 86% for all patients (*n* = 24) (Table 3). No significant association was found between PAR-index and satisfaction with treatment (Table 4). The same result was observed when the two patients who underwent only orthognathic surgery (Le Fort 1 osteotomy and with an acceptable dental occlusion) without orthodontic treatment were excluded (data not shown). Therefore, we included these two patients in the final analysis.

**Table 3:** Distribution of median PAR-scores and change in patients going through orthognathic surgery at the University Hospital of North-Norway in 2016–2025 (*n* = 24).

	Pre-weighted PAR-score median (IQR)	Post-weighted PAR-score median (IQR)	Change in PAR-score median (IQR)	*P*-value	Change (%) median (IQR)	PAR-index median (IQR)
Total (*n* = 22)	26.5 (18.5–36.3)	4.0 (0.8–8.0)	19.0 (10.8–32.0)	<0.001*	85.5 (68.8–100.0)	1.5 (1.0–2.0)

PAR-index, the peer assessment rating index; 1 Great improvement >70%; 2 Improvement 30%–70%; 3 Worse or no difference 0%–30%; *P* < 0.05 for statistical significance.

* Wilcoxon signed rank test.

**Table 4:** Correlation between PAR-index and satisfaction with treatment in patients median 28 months postoperative orthognathic surgery (*n* = 24).

	Satisfaction with dental occlusion*		Satisfaction with facial esthetics*		Satisfaction with TMJ-function*	
PAR-index	Spearman's rho	*P*-value	Spearman's rho	*P*-value	Spearman's rho	*P*-value
	−0.14 *n* = 22	0.53	−0.05 *n* = 22	0.82	−0.31 *n* = 22	0.16

PAR-index, the peer assessment rating index; 1 Great improvement >70%; 2 Improvement 30%–70%; 3 Worse or no difference 0%–30%; *P* < 0.05 for statistical significance and crosstabulation spearman's rho; *Satisfaction score; 1 Dissatisfied; 2 Not so satisfied; 3 Neutral; 4 Satisfied; 5 Very satisfied.

Most patients were either satisfied or very satisfied with the treatment (61%–86%) (Table 5). Eight females [8/24 (33%)] were either dissatisfied or less satisfied with the treatment, especially healing time and total treatment time: 2/24 (8%) with facial esthetics and 2/24 (8%) with TMJ-function after surgery, 5/24 (21%) with healing period after surgery, and finally 4/24 (17%) with total treatment time. No correlation was found between age and satisfaction with treatment.

**Table 5:** Satisfaction (=satisfied or very satisfied) with treatment in patients median 28 months postoperative orthognathic surgery (*n* = 24).

Satisfaction with treatment	Number (%)
Facial esthetics	19/22 (86)
Dental occlusion	19/22 (86)
TMJ-function	19/23 (83%)
Healing time	14/23 (61%)
Total treatment time	14/22 (64%)
Psychological assessment	19/22 (86%)

### Cephalometric assessments

Pre- and postoperative cephalometric variables according to malocclusion/deformity are presented in Table 6. In class II malocclusion, the ML-NSL angle was significantly changed (P 0.054) median 28 months after surgery. In class III malocclusion, the following angles were significantly changed median 28 months after surgery: ANB (*P* = <0.001), Wits (*P* = 0.009). Degree of proclination and retroclination of incisors did not change significantly (i.e., degree of decompensation).

**Table 6:** Cephalometric variables according to deformity in patients pre- and median 28 months postoperative orthognathic surgery at UNN in 2016–2025 (*n* = 24).

Cephalometric variable	Deformity CL I (*n* = 9) CL II (*n* = 5) CL III (*n* = 7)	Preop mean (SD)	Postop mean (SD)	Difference mean (SD)	*P*-value*
ANB	CL I	2.8 (1.2)	2.0 (1.6)	0.8 (2.0)	0.31
	CL II	6.7 (2.1)	4.7 (4.3)	2.0 (2.2)	0.17
	CL III	−3.6 (2.2)	−0.2 (2.7)	−3.5 (1.2)	<0.001
Wits	CL I	−9.2 (28.6)	−1.8 (2.9)	−7.4 (28.1)	0.48
	CL II	6.4 (1.2)	3.1 (2.8)	3.4 (3.7)	0.16
	CL III	−9.1 (2.5)	−5.3 (0.5)	−3.7 (2.2)	0.009
ML-NSL	CL I	33.0 (7.2)	33.2 (6.5)	−0.2 (3.6)	0.87
	CL II	27.9 (4.5)	30.5 (4.1)	−2.6 (1.7)	0.054
	CL III	33.5 (8.7)	32.7 (6.7)	0.9 (4.0)	0.62
Li-NB	CL I	29.4 (9.3)	30.7 (5.0)	−1.3 (7.2)	0.64
	CL II	29.0 (5.0)	31.6 (4.2)	−2.6 (4.0)	0.29
	CL III	18.7 (7.9)	17.7 (7.6)	1.0 (3.0)	0.45
Li-ML	CL I	98.3 (9.8)	98.9 (8.0)	−0.6 (6.6)	0.82
	CL II	105.2 (2.7)	104.8 (1.2)	0.5 (3.3)	0.80
	CL III	82.0 (7.2)	81.9 (8.5)	0.1 (4.7)	0.96

ANB, the angle formed by point A, nasion (N) and point B; Wits, wits appraisal; ML-NSL, the angle formed by the mandibular line and Nasion-Sella-Line; Li-NB, the inclination of the lower incisors in relation to the nasion-point B line; Li-ML, the angle between the long axis of the lower central incisor and mandibular plane; *P* < 0.05 for statistical significance.

* Paired sample t-test.

### Psychological assessments

The median time between preoperative psychological consultation and surgery was 18 months, while the median time between postoperative psychological consultation and surgery was 28 months (Table 1). HADS and SWLS did not change significantly after surgery (Table 7). No significant gender differences were found for HADS or SWLS scores. There was no significant correlation between preoperative HADS and satisfaction with treatment. However, higher scores on SWLS correlated negatively with satisfaction with dental occlusion (*P* = 0.020) (Table 8).

**Table 7:** HADS and SWLS scores in patients going through orthognathic surgery at the University Hospital of North-Norway in 2016–2025 (*n* = 24).

	Preop median (IQR)	Postop median (IQR)	*P*-value
HADS	8.5 (4.0–11.8) (*n* = 22)	10.0 (5.0–13.8)	0.614*
SWLS	23.5 (18.0–29.0)	24.5 (21.0–31.0)	0.455*

HADS, the hospital anxiety and depression scale; SWL, the subjective wellbeing of Life Scale; *P* < 0.05 for statistical significance.

* Wilcoxon signed rank test.

**Table 8:** Correlation between preoperative HADS, SWLS and satisfaction with treatment median 28 months postoperative orthognathic surgery (*n* = 24).

	Satisfaction with dental occlusion*		Satisfaction with facial esthetics*		Satisfaction with TMJ-function*	
	Spearman's rho	*P*-value	Spearman's rho	*P*-value	Spearman's rho	*P*-value
HADS	0.21 *n* = 20	0.37	0.01 *n* = 20	0.98	0.04 *n* = 21	0.87
SWLS	−0.49 *n* = 22	**0.02**	−0.09 *n* = 22	0.69	−0.27 *n* = 23	0.22

HADS, the hospital anxiety and depression scale; SWL, the subjective wellbeing of Life Scale; *P* < 0.05 for statistical significance; *Satisfaction score; 1 Dissatisfied; 2 Not so satisfied; 3 Neutral; 4 Satisfied; 5 Very satisfied.

## Discussion

In this prospective quality assurance study, all patients were assessed by a psychologist before and after orthognathic surgery. Dental and skeletal deformity improved after surgery, but dental occlusion did not correlate with satisfaction with treatment. Complications were frequent but mild in severity. HADS and SWLS were within normal range before surgery and did not change significantly after surgery. HADS did not correlate to satisfaction with treatment, but higher scores in SWLS before surgery correlated negatively with satisfaction with dental occlusion after surgery. Most patients were satisfied with the treatment outcome, except for the overall duration of the treatment and the healing period after surgery.

About one fifth of the patients had facial asymmetry in our study. Of these, 50% had either juvenile idiopathic arthritis (JIA) or rheumatic arthritis (RA). The incidence of JIA is highest in the northern regions of Norway (19). TMJ arthritis in JIA can lead to growth disturbances with dentofacial deformity (3), such as facial asymmetry, class II relation, micrognathia with anterior open bite and a reduction in posterior airway space and related comorbidities such as sleep apnea (4) and reduced quality of life (20). Facial asymmetry is often treated with bimaxillary orthognathic surgery, with or without counterclockwise rotation, and appears to be highly stable despite the potential for relapses (21).

Treatment outcomes such as dental occlusion and skeletal measurements improved after treatment, consistent with other studies on orthognathic surgery using the PAR-index scores for measurement (22, 23). A high-quality occlusal outcome is essential for all patients, as achieving proper intercuspation is believed to enhance long-term stability (24). The difference between the pre- and post-treatment scores in PAR reflects the degree of improvement of treatment (18). In our study, reduction in PAR score (“improvement”) before and after treatment was in total 86%. However, in two patients who underwent surgical correction of facial asymmetries, no change was seen in dental occlusion because orthodontic treatment was not performed; only a clockwise surgical procedure was carried out. In these cases, the PAR index is not a suitable measure of improvement and by eliminating these patients the percentage of improvement would have been even higher.

We did not find a correlation between PAR-index and satisfaction with treatment. A reduction in the PAR-index score (i.e., better dental occlusion) is often positively correlated with a better subjective outcome measured by oral health-related quality of life and satisfaction with treatment (25). However, Uppada et al. emphasize that surgeons assessments of a successful outcome do not always align with patients perceptions (26), which is consistent with our findings. This underlines the advantage of psychological assessment before surgery to better explore the patient's expectations.

Most patients in our study reported satisfaction with dental and facial appearance (86%) after surgery. Patient satisfaction after orthognathic surgery is generally high, ranging from 83% in the Torgersbråten et al. study (27) to 100% in Pahkala et al.'s study (28). We found that in total 33% were dissatisfied or less satisfied with certain aspects of the treatment. Dissatisfaction with healing period after surgery and total treatment time were most common, which is in line with Pachêco-Pereira et al. (29) who reported that factors associated with dissatisfaction were treatment length; sensation of functional impairment and/or dysfunction after surgery, and perceived omitted information about surgical risks. We believe that the best way to improve satisfaction is through preoperative examinations, communication of goals, and realistic expectations. Addressing psychological factors and providing support can also play an essential role in improving satisfaction levels after surgery.

Complications were frequent. Despite one serious adverse event (infection) requiring prolonged hospitalization, most complications were mild, such as neurosensory disturbances. Neurosensory disturbance of the inferior alveolar nerve is a common complication following BSSO in the immediate postoperative period and higher age is reported to be a risk factor. However, nerve function typically recovers over time in the long term (30), even if sensation may be altered. Espeland et al. reported that 37% had altered sensation at 3-year follow-up after orthognathic surgery (2).

We found that HADS-total (preop median 8.5) and SWLS (preop median 23.5) scores were within normal range before surgery and did not change significantly after surgery (HADS median 10,0, SWLS 24.5) (15, 16). The fact that the long lasting and demanding treatment did not lead to a worsening in psychological health is noteworthy. We cannot rule out that the extra support our patients received in terms of consultations with a psychologist might have provided a buffer against distress and worsening psychological symptoms.

Among the studies on orthognathic surgery and quality of life using HADS, Lin et al. (31), assessed 77 patients receiving orthognathic surgery and 32 age and gender-matched controls according to HADS, and in line with our study no significant differences were found between the groups, nor between before and after surgery. Several specific and validated quality of life questionnaires are available for assessing outcomes in orthognathic surgery, such as the Orthognathic Quality of Life Questionnaire (OQLQ) (32, 33). However, it has not yet been translated or adapted for use in the Norwegian language.

Our study did not find a correlation between HADS and satisfaction with treatment, only a significant negative correlation between SWLS and satisfaction with dental occlusion was found. One possible interpretation is that individuals with higher levels of overall life satisfaction may also hold higher expectations regarding treatment outcomes, in this case dental occlusion. When expectations exceed what can realistically be achieved, this may contribute to reduced perceived satisfaction following treatment. Although causal inferences cannot be drawn from the current findings, the results suggest that baseline expectations may be a relevant factor in how patients evaluate outcomes. Addressing and aligning expectations prior to surgery may therefore be of importance for optimizing perceived treatment satisfaction.

Few studies about orthognathic surgery include prospective patient-related outcomes evaluating psychological status and satisfaction with validated screening tools such as HADS and SWLS, which are strengths for this study. However, the study-specific questionnaires used to investigate satisfaction with the outcome and process were not based on any internationally validated scale, which is a limitation of our study. Other limitations with the study were recall and selection biases more than one year after surgery, confounding variables that are hard to control for, and missing data due to reliance on existing records. Also, the variability in timing between assessments may be a confounder, as differences in follow-up intervals may have influenced psychological outcomes independently of the surgical intervention. Our results must also be evaluated in the context of the low sample size of surgical cases included in the study, making analyses of multiple correlations and subgroups such as gender and age less reliable, and this should be reflected in a cautious interpretation of the findings. In addition, the inclusion of only 24 patients with complete pre- and postoperative psychological assessments may introduce a degree of selection bias.

## Conclusion

Treatment outcomes such as dental occlusion and skeletal deformity improved after orthognathic surgery and complications were frequent but mild. Dental occlusion did not correlate to satisfaction with treatment. HADS and SWLS scores were within the normal range among patients before surgery and did not change significantly after surgery. Nevertheless, routine psychological and clinical assessments remain crucial for identifying individuals at risk of developing clinically significant symptoms in the future and ensuring they receive appropriate support. Most patients were satisfied with the treatment outcome, except for overall duration of the treatment and the healing period after surgery. Therefore, we emphasize the importance of providing thorough information prior to surgery regarding postoperative pain, discomfort, and total treatment time.

## Data Availability

The raw data supporting the conclusions of this article will be made available by the authors, without undue reservation.
